# Novel Insights into the PKCβ-dependent Regulation of the Oxidoreductase p66Shc*

**DOI:** 10.1074/jbc.M116.752766

**Published:** 2016-09-13

**Authors:** Martina Haller, Sana Khalid, Leopold Kremser, Friedrich Fresser, Tobias Furlan, Martin Hermann, Julia Guenther, Astrid Drasche, Michael Leitges, Marco Giorgio, Gottfried Baier, Herbert Lindner, Jakob Troppmair

**Affiliations:** From the ‡ Daniel Swarovski Research Laboratory, Department of Visceral, Transplant, and Thoracic Surgery,; § Division of Clinical Biochemistry, Protein Micro-Analysis Facility,; ¶ Department for Pharmacology and Genetics, Division of Translational Cell Genetics, and; ‖ Department for Anesthesiology and Intensive Care, Medical University of Innsbruck, 6020 Innsbruck, Austria,; the ** Biotechnology Center of Oslo, 0349 Oslo, Norway, and; the ‡‡ European Institute of Oncology, 20139 Milano, Italy

**Keywords:** cell death, phosphotyrosine binding (PTB) domain, PKC, reactive oxygen species (ROS), redox signaling, p66shc, phosphorylation

## Abstract

Dysfunctional mitochondria contribute to the development of many diseases and pathological conditions through the excessive production of reactive oxygen species (ROS), and, where studied, ablation of p66Shc (p66) was beneficial. p66 translocates to the mitochondria and oxidizes cytochrome *c* to yield H_2_O_2_, which in turn initiates cell death. PKCβ-mediated phosphorylation of serine 36 in p66 has been implicated as a key regulatory step preceding mitochondrial translocation, ROS production, and cell death, and PKCβ thus may provide a target for therapeutic intervention. We performed a reassessment of PKCβ regulation of the oxidoreductase activity of p66. Although our experiments did not substantiate Ser^36^ phosphorylation by PKCβ, they instead provided evidence for Ser^139^ and Ser^213^ as PKCβ phosphorylation sites regulating the pro-oxidant and pro-apoptotic function of p66. Mutation of another predicted PKCβ phosphorylation site also located in the phosphotyrosine binding domain, threonine 206, had no phenotype. Intriguingly, p66 with Thr^206^ and Ser^213^ mutated to glutamic acid showed a gain-of-function phenotype with significantly increased ROS production and cell death induction. Taken together, these data argue for a complex mechanism of PKCβ-dependent regulation of p66 activation involving Ser^139^ and a motif surrounding Ser^213^.

## Introduction

Damage caused by reactive oxygen species contributes to the onset and progression of many diseases and pathological conditions, including diabetes, neurodegeneration, ischemia/reperfusion injury (IRI),^3^ stroke, and cardiovascular diseases (1). The use of antioxidants, *e.g.* for the prevention of IRI, has little benefit (2–7), most likely because of the failure to efficiently and timely target these substances to the site of ROS production and action. ROS produced at the mitochondria are normally the byproduct of the incomplete reduction of O_2_ in the electron transport chain (ETC). In particular, complexes I and III are involved in this process (8). Work published over the last years has suggested that mitochondrial processes, including ROS production, are subject to regulation by intracellular signaling (9–12). Phosphorylation of mitochondrial proteins, including subunits of cytochrome *c* oxidase (COX, complex IV) by PKA modulated ATP generation and ROS production (12). Similarly, phosphorylation of complex I of the ETC by PKA led to decreased ROS production (13). Lowered mitochondrial ROS levels were observed in cells expressing the survival proteins RAF, AKT, and Bcl-2 (14), whereas p38MAPK has been implicated in causing redox stress (15–17). PKCϵ protects cells against stress and regulates the ETC, controlling the processes of respiration and ROS production (18, 19). Evidence has also been provided for the cardioprotective effect of directly inhibiting complex I activity (20). Interfering with signaling pathways, which are activated during cellular stress and control mitochondrial function, thus may become an alternative approach for the prevention of oxidative damage.

A direct role in mitochondrial ROS production has been demonstrated for p66 (21). p66 is a redox enzyme that generates H_2_O_2_ through the oxidation of cytochrome *c* (21). p66-deficient mice lacked any defects during development or adult life but showed, on average, a 30% prolongation of their life span, which correlated with increased resistance to oxidative stress because of a decreased production of ROS, whereas scavenging systems were not affected (21). p66 ablation in mice was beneficial in many disease settings caused by oxidative stress (22–30). These observations suggest that p66 may have a key function in the cellular response to stress. Because of the lack of inhibitors of its oxidoreductase function, interfering with its upstream activity pathway holds potential for the development of novel therapeutic approaches for the prevention of clinical conditions associated with excessive production of ROS.

PKCβ has been proposed as an important regulator of the pro-oxidant and pro-apoptotic function of p66 (31). In particular, the phosphorylation of serine 36 on p66 is required for Pin1 binding and mitochondrial import (31). In this work, we further studied the regulation of p66 by PKCβ. The existence of additional regulatory phosphorylation sites was suggested by the observation of a pronounced effect of PKCβ inhibition on ROS production after pro-oxidant exposure, whereas Ser^36^ phosphorylation was not affected. Moreover, the site surrounding Ser^36^ conforms to a MAPK phosphorylation motif and may be targeted by p38, JNK, and ERK (32, 33). Phosphorylation of p66 on Ser^36^ has been reported in response to various stress stimuli (29–31, 34–38). We have shown recently that JNK1/2 are involved in the phosphorylation of this site and of p66 pro-oxidant and pro-death function (39). Here we describe the identification and functional characterization of three novel PKCβ phosphorylation sites on p66 that are required for mitochondrial ROS production and apoptosis induction in response to oxidative stress. These data, together with our recent demonstration of the p66 Ser^36^ kinase activities of JNK1/2 (39), demonstrate that the regulation of p66 activation is more complex than previously anticipated. Our results also support that targeting upstream kinases involved in the activation of p66 during cellular stress offers a novel approach for the prevention of oxidative damage.

## Results

### 

#### 

##### Regulation of p66 Activation by PKC

**FIGURE 1.**
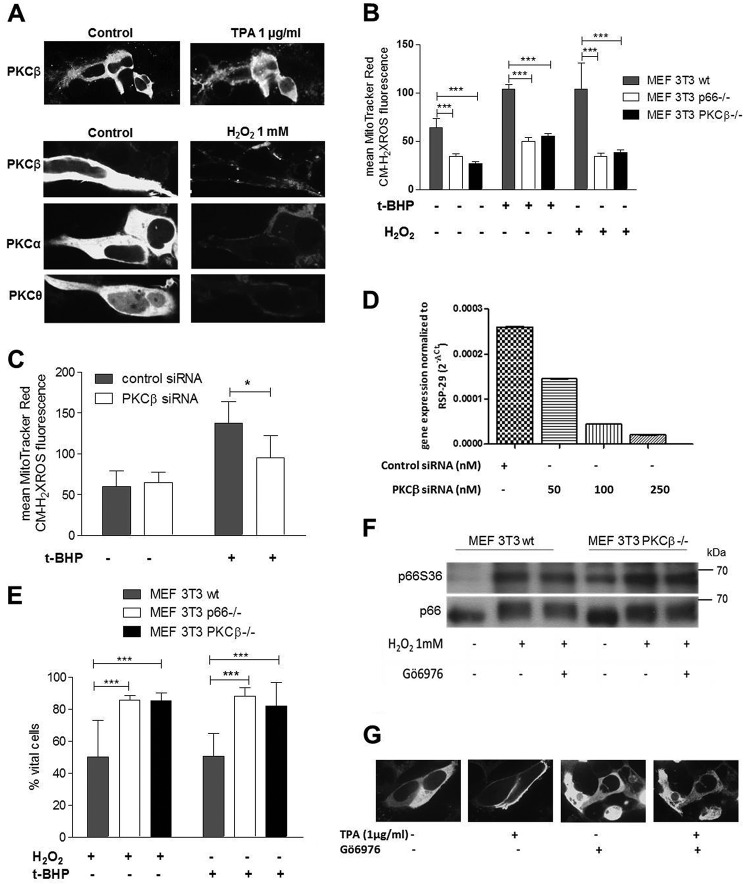
**Regulation of p66 redox activity by PKC.** *A*, HEK293 cells were transfected with pECFP-PKCα, β, or θ, and fluorescence images were acquired before and after treatment with TPA (1 μg) or an oxidative challenge (H_2_O_2_ 1 mm). Before stress application, all PKC isoforms showed a predominantly cytosolic distribution as described previously (31). Upon TPA stimulation or H_2_O_2_ treatment, distinct membrane fluorescence was detected (*n* ≥ 3). All images were acquired using a ×63 oil immersion objective, and for better visualization, the images were adjusted, and representative cells are shown. *B*, MEF 3T3 WT, p66^−/−^, and PKCβ^−/−^ cells were treated with *t*-BHP or H_2_O_2_ (1 mm, 30 min) (*n* ≥ 4). *C*, effect of PKCβ knockdown on mitochondrial ROS levels in MEF 3T3 WT cells after *t*-BHP exposure (1 mm, 30 min) (*n* = 4). *D*, PKCβ expression was determined by real-time quantitative PCR after transfecting cells with PKCβ or control siRNA and normalized to the housekeeping gene *RSP-29. E*, MEF 3T3 WT, p66^−/−^, and PKCβ^−/−^ cells were treated for 24 h with 800 μm H_2_O_2_, 30 μm *t*-BHP. The percentage of vital cells was determined after Annexin V/propidium iodide staining (*n* ≥ 3). *F*, H_2_O_2_ (1 mm, 30 min) induced p66 phosphorylation on Ser^36^ in both PKCβ 3T3 WT and PKCβ^−/−^ MEFs, whereas preincubation with Gö6976 (500 nm, 1 h) did not abrogate p66 Ser^36^ phosphorylation (*n* ≥ 3). *G*, pretreatment of PKCβ-CFP-transfected HEK293 cells with Gö6976 (500 nm) prevents PKCβ plasma membrane translocation. Statistics were done using ANOVA (*, *p* < 0.05; ***, *p* < 0.001).

To define the requirement of PKCβ for ROS production, we first confirmed that the experimental conditions studied here result in the activation of PKC, monitored by cell membrane translocation of PKC, as shown before (31). 12-*O*-tetradecanoylphorbol-13-acetate (TPA), a potent PKC activator, was used as a positive control. We observed cell membrane translocation of all isoforms tested (PKCα, β, and θ) in the case of pro-oxidant treatment in HEK293 cells (Fig. 1*A*) as well as in mouse embryonic fibroblasts (MEFs) (data not shown). Next we analyzed PKCβ-deficient immortalized MEFs and measured ROS levels 30 min after treatment with the pro-oxidants H_2_O_2_ or *t*-BHP (40). PKCβ-deficient cells showed decreased mitochondrial ROS production after pro-oxidant treatment, as monitored by MitoTracker Red CM-H_2_XRos (or 2′,7′-dichlorodihydroflourescence (DCF), data not shown) fluorescence, which was comparable with the response observed in cells lacking p66 (Fig. 1*B*). Expression of PKCβ in PKCβ^−/−^ MEFs restored basal and pro-oxidant-induced ROS levels (data not shown). Conditional knockdown of PKCβ in WT MEF 3T3 cells using siRNA further confirmed its role in stress-induced ROS production (Fig. 1*C*). siRNA knockdown of PKCβ was confirmed with quantitative real-time PCR (Fig. 1*D*). Furthermore, PKCβ^−/−^ cells showed increased resistance to cell death induced by pro-oxidants, as reported for p66^−/−^ cells (41) (Fig. 1*E*).

Given the importance of Ser^36^ phosphorylation for p66 activation, we analyzed PKCβ-deficient and WT MEFs for the presence of this posttranslational modification. We observed in our experiments phosphorylation of p66 on Ser^36^ in WT MEFs after 30 min of treatment with H_2_O_2_ (Fig. 1*F*) or *t*-BHP (data not shown), but we were unable to reduce or prevent Ser^36^ phosphorylation by treatment with two different PKC inhibitors, Gö6850 (data not shown) and Gö6976 (Fig. 1*F*). Inhibition of PKCβ with Gö6976 was confirmed because it reduced cell membrane translocation of PKCβ upon TPA treatment (Fig. 1*G*) as well as after pro-oxidant treatment both in HEK293 cells and in MEFs (data not shown). PKCβ-deficient MEFs showed slightly increased Ser^36^ phosphorylation but, like their WT counterpart, responded with further increased phosphorylation to H_2_O_2_ treatment (Fig. 1*F*). However, because PKCβ inhibition, as well as PKCβ knockdown and knockout, was very effective in lowering mitochondrial ROS levels in response to pro-oxidant treatment without decreasing Ser^36^ phosphorylation, we hypothesized that Ser^36^ might not be the critical PKCβ-regulated site on p66.

##### Ser^139^, Thr^206^, and Ser^213^ Are PKCβ-targeted Sites on p66

**FIGURE 2.**
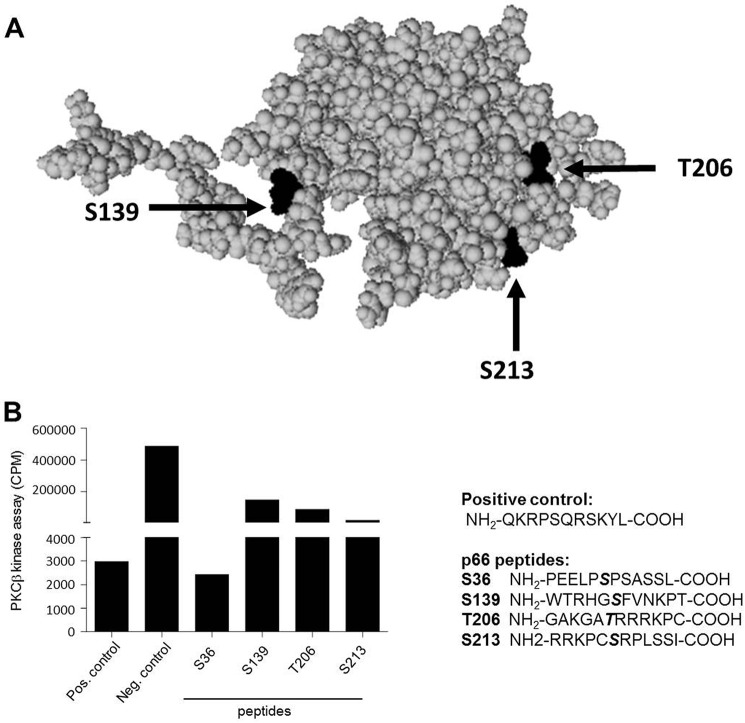
**PKCβ regulatory sites in p66.** *A*, PyMOL was used to visualize and inspect the structure of the PTB domain of the Shc1 protein harboring Ser^139^, Thr^206^, and Ser^213^. Structural information obtained from the PDB (1SHC) was obtained for the smaller p52Shc isoform, and the numbering of amino acids, therefore, had to be adjusted accordingly. Thus, Ser^139^, Thr^206^, and Ser^213^ correspond to Ser^29^, Thr^96^, and Ser^103^, respectively. *B*, recombinant PKCβ was used to phosphorylate peptides containing Ser^36^, Ser^139^, Thr^206^, or Ser^213^. *Pos*, positive; *Neg*, negative; *CPM*, counts per minute.

A scan for potential PKC phosphorylation sites (42) in p66 predicted three amino acid residues: Ser^139^, Thr^206^, and Ser^213^ (43). These predicted phosphorylation sites lie within (Thr^206^ and Ser^213^) or adjacent to (Ser^139^) the phosphotyrosine binding (PTB) domain of p66 (21). The structure of the PTB domain of Shc1 protein has been resolved by NMR (44, 45) (PDB code 1SHC) and x-ray diffraction (PDB code 4XWX), and visual inspection suggests that these three sites are located on the surface of the protein (Fig. 2*A*) and thus freely accessible for the kinases. We confirmed the predicted PKCβ phosphorylation sites by using *in vitro* kinase assays with recombinant PKCβ and peptides harboring any one of these three predicted sites. We could show phosphorylation of peptides containing Ser^139^, Thr^206^, or Ser^213^, but we were not able to detect substantial Ser^36^ phosphorylation (Fig. 2*B*). These results are in agreement with our previous observation where we identified JNK1/2 as p66 Ser^36^ kinases (39).

**FIGURE 3.**
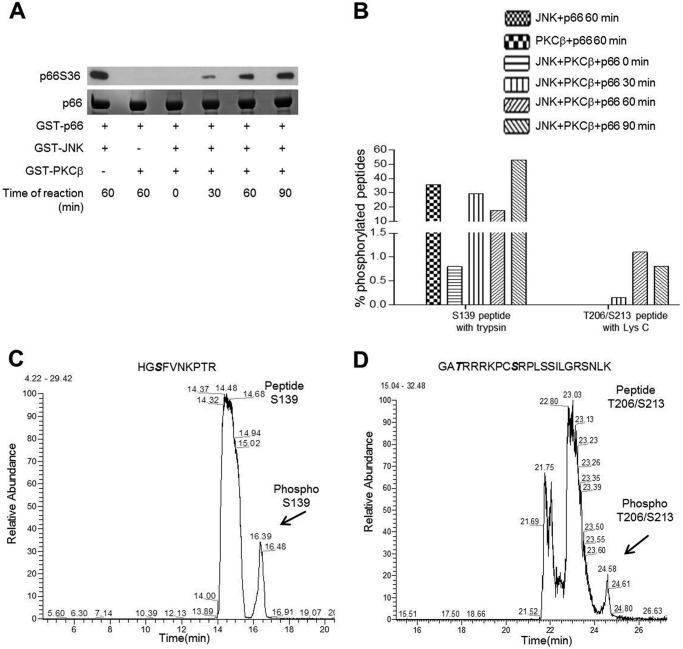
**Ser^139^, Thr^206^, and Ser^213^ are PKCβ-regulatory sites.** *A*, for mass spectrometry, an *in vitro* kinase assay was performed with recombinant kinases and p66 as substrate and subjected to SDS-PAGE. The gel was stained with Coomassie or immunoblotted for p66 Ser^36^. *B*, the protein bands were excised from Coomassie-stained gel and digested with trypsin or Lys C. Both Tryptic (*C*) and Lys C (*D*) digests were analyzed by nano-HPLC coupled via an electrospray ionization interface to a Q Exactive HF mass spectrometer. Data analysis and peak area calculation were performed using Proteome Discoverer 1.4.1.14.

Next we confirmed these three sites in an *in vitro* kinase assay by using full-length recombinant p66 protein. The protein was incubated with JNK1 or PKCβ kinase alone or in combination under kinase assay conditions. The reaction products were separated by SDS-PAGE, which was further immunoblotted to detect p66 Ser^36^ (Fig. 3*A*) or stained with Coomassie. GST-Shc was excised from the gel and digested either with trypsin or Lys C to yield peptides containing Ser^139^ and Thr^206^/Ser^213^, respectively, detectable by MS. As shown in Fig. 3*B*, incubation with PKCβ resulted in a time-dependent increase in the number of p66 Ser^139^-phosphorylated peptides alone or in combination with JNK but not with JNK alone. p66 Ser^36^ phosphorylation was observed with JNK alone or in combination with PKCβ but not with PKCβ alone (Fig. 3*A*). However, Thr^206^- and Ser^213^-phosphorylated peptides were only detected when both kinases were used together (Fig. 3, *B–D*). The percentage of Thr^206^/Ser^213^-containing phosphopeptides detected was significantly lower than that of Ser^139^-phosphorylated peptides.

**FIGURE 4.**
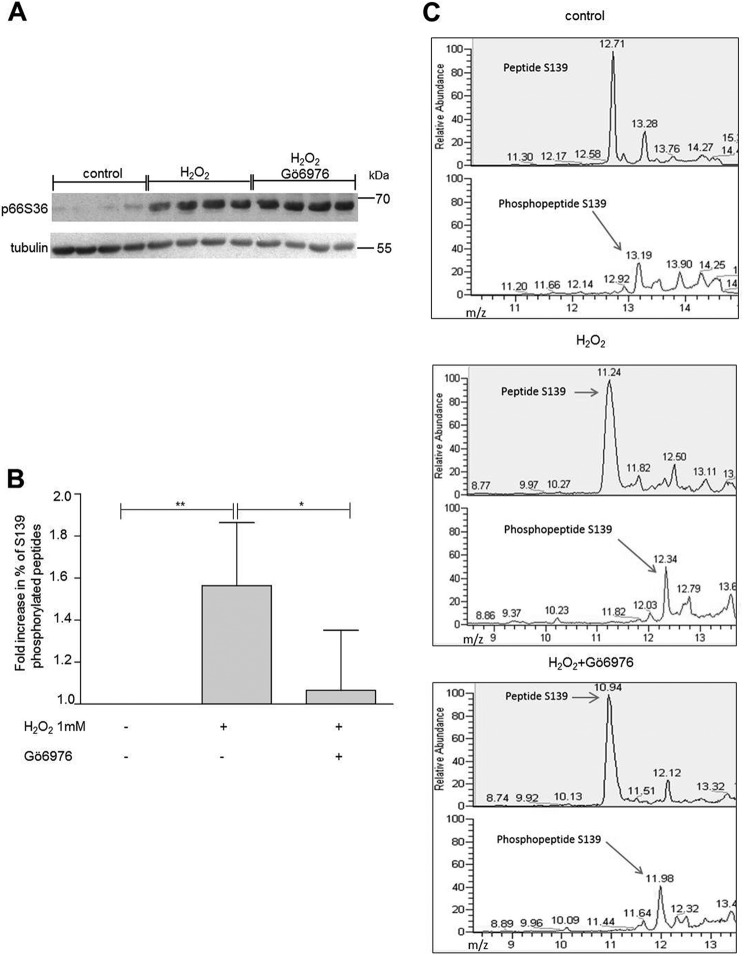
**Cellular phosphorylation of Ser^139^ by PKCβ.** *A*, p66 overexpressed in HEK293 cells and stressed with 1 mm H_2_O_2_ for 15 min either alone or in the presence of Gö6976. Cells lysates were analyzed for p66 Ser^36^ phosphorylation by Western blotting or immunoprecipitated for mass spectrometry. *B* and *C*, proteins were digested on beads with trypsin and analyzed by nano-LC coupled via an electrospray ionization interface to a Velos mass spectrometer. The amounts of phosphorylated and non-phosphorylated peptide were calculated by the peak heights of the extracted ion chromatograms provided. A summary graph of more than three individual biological experiments and mass spectrometry analyses is provided. Statistical significance was determined by using ANOVA (*, *p* < 0.05; **, *p* < 0.01).

In our cellular assays, increased p66 Ser^36^ phosphorylation upon pro-oxidant treatment was not affected by the PKC inhibitor Gö6976 (Fig. 4*A*), which efficiently decreased Ser^139^ phosphorylation that is detected by MS (Fig. 4, *B* and *C*). However, in contrast to the *in vitro* experiments (Fig. 3, *B–D*), no Thr^206^/Ser^213^ phosphorylation was detected.

##### The Role of Ser^139^ in PKCβ-mediated Activation

**FIGURE 5.**
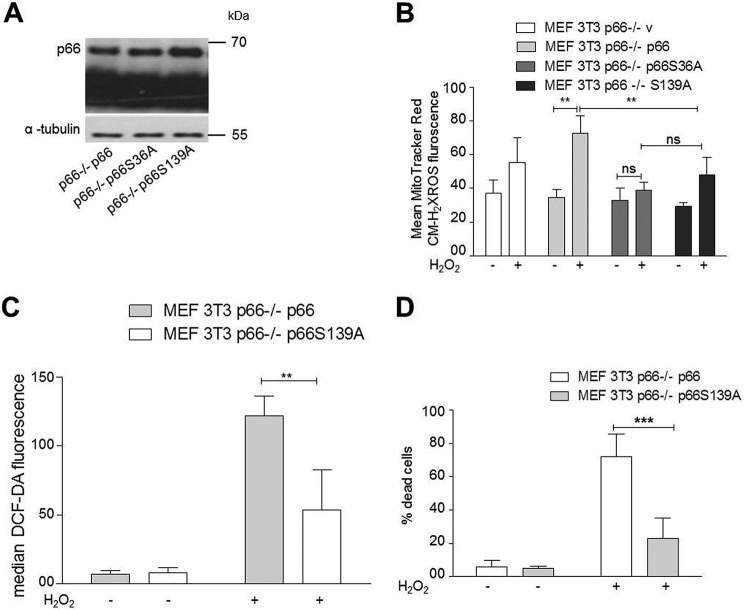
**Ser^139^ in p66 contributes to ROS and cell death regulation upon stress.** *A*, wild-type p66 or the mutants p66S36A and p66S139A were transfected in p66Shc^−/−^ 3T3 MEFs in triplicates and selected for 2 weeks with puromycin (4 μg/ml). Cells were lysed and checked for protein expression with immunoblotting, and cells showing equal expression were scaled up for further experiments. *B*, cells were treated for 30 min with 1 mm H_2_O_2_. Mitochondrial ROS levels were detected by staining the cells with MitoTracker Red CM-H_2_XROS and visualized by fluorescent microscope (*n* ≥ 3) or (*C*) by staining the cells with DCF-DA, which was measured via FACS. *D*, cell death of MEFs expressing WT p66 or p66 mutated in Ser^139^ after 24-h treatment with 500 μm H_2_O_2_ (*n* ≥ 5). Statistical significance was determined using *t* test or ANOVA (**, *p* < 0.01; ***, *p* ≤ 0.001; *n.s.*, not significant).

Ser^139^ in p66, equivalent to Ser^29^ in p52Shc, has already been reported to be phosphorylated by the PKCα, ϵ, and δ isoforms and is essential for the interaction of ShcA with the protein tyrosine phosphatase protein tyrosine phosphatase PEST (PTP-PEST) (43). However, a role in pro-oxidant signaling by PKCβ/p66 has never been analyzed. To address the function of Ser^139^ in p66 activation/activity, we reconstituted p66-deficient cells with either the S36A or S139A mutant of p66 or with WT p66 and measured ROS production following H_2_O_2_ treatment. Equal expression of p66 and its mutants was confirmed by immunoblotting (Fig. 5*A*). As shown in Fig. 5, *B* and *C*, the alanine exchange caused significantly reduced ROS production upon stress. We were also able to show that reduced ROS production in p66S139A reconstituted MEFs correlated with decreased cell death (Fig. 5*D*).

##### Thr^206^ and Ser^213^ in p66 Are Critical for the Control of ROS Production and Cell Death by PKCβ

**FIGURE 6.**
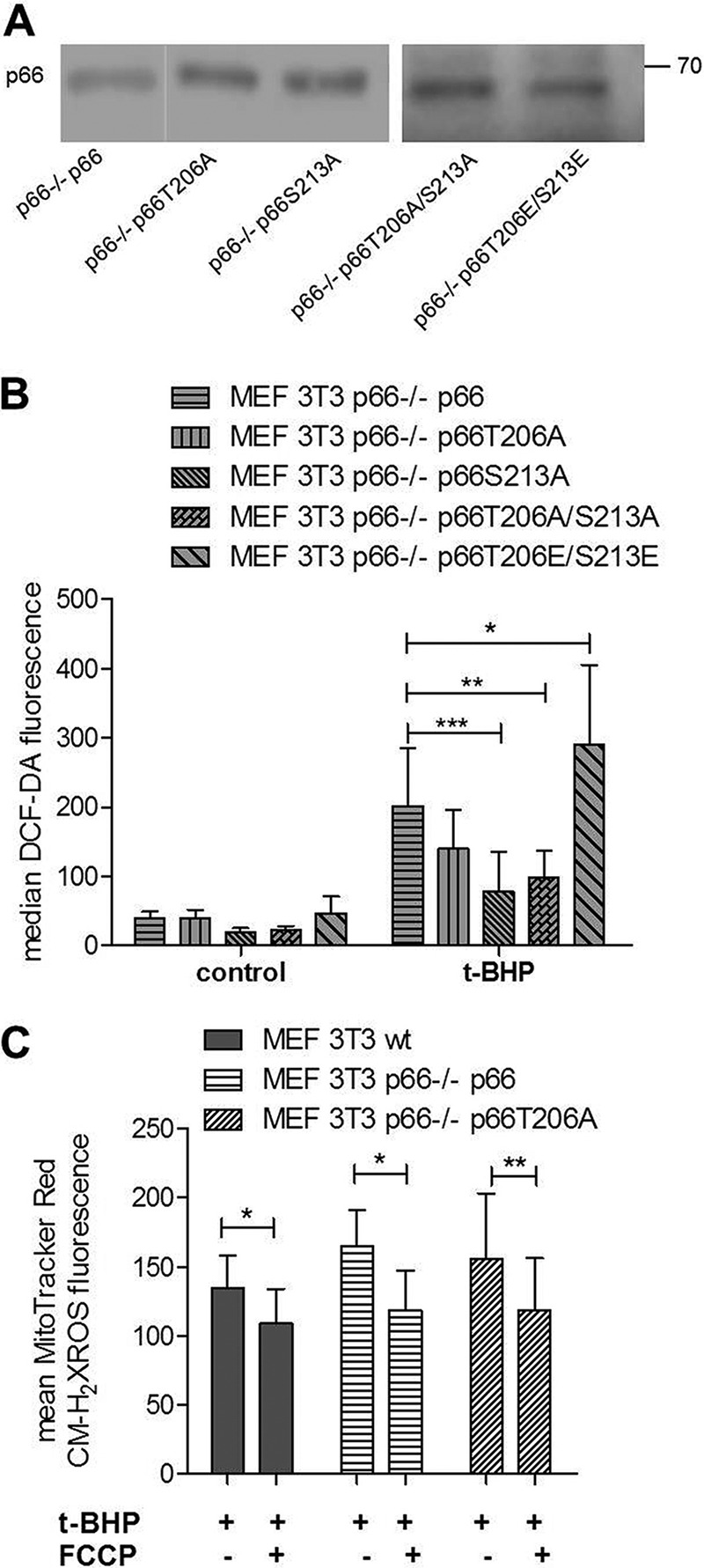
**Ser^213^ in p66 is critical for ROS production.** Wild-type p66 or the mutants T206A, S213A, T206/S213A, and T206E/S213E were transfected in p66Shc^−/−^ 3T3 MEFs in triplicates and selected for 2 weeks with puromycin (4 μg/ml). *A*, cells were lysed and checked for protein expression with immunoblotting, and cells showing equal expression were scaled up for further experiments. The results shown in individual panels were run on the same blot and cropped for clarity. *B*, ROS levels of p66^−/−^ MEFs expressing wild-type p66 or p66 mutated in Thr^206^ and/or Ser^213^ in full-serum medium (control) after 30 min of *t*-BHP treatment (1 mm) (*n* ≥ 4). *C*, mild uncoupling with carbonyl cyanide *p*-trifluoromethoxyphenylhydrazone (*FCCP*) (5 μm, 15 min preincubation) decreased mitochondrial ROS levels in MEFs expressing wild-type or Thr^206^-mutated p66 (*n* ≥ 5). Statistical analysis was performed using either *t* test or analysis of variance (*, *p* < 0.05; **, *p* < 0.01; ***, *p* < 0.001).

The physiological relevance of the two PKCβ phosphorylation sites was evaluated following stable expression of mutant p66 proteins in p66^−/−^ MEFs. Equal protein expression was confirmed by immunoblotting (Fig. 6*A*). Two different phenotypes were observed (Fig. 6*B*). Although mutating Thr^206^ did not affect the ability of p66 to produce ROS, maintaining the integrity of Ser^213^ was essential for the pro-oxidant function of p66 both under basal growth conditions and in response to H_2_O_2_ treatment (Fig. 6*B*). Additional mutations tested included the individual exchange of these residues to glutamic acid. However, these mutants did not behave differently from the alanine mutants for all parameters studied here (data not shown). Exchanging Thr^206^ and Ser^213^ to alanine resulted in a protein that did not differ from S213A (Fig. 6*B*). In contrast, replacing both residues with glutamic acid created a protein that was hyperactive with regard to ROS production (Fig. 6*B*). This, to our knowledge, is the first demonstration of a constitutively active form of p66. To assure that mutant p66 specifically affected mitochondrial ROS production, we treated cells with low concentrations of the uncoupler carbonyl cyanide *p*-trifluoromethoxyphenylhydrazone. Such mild uncoupling of mitochondria is known to decrease mitochondrial ROS production (14). As shown in Fig. 6*C*, this treatment significantly reduced p66-dependent ROS production.

**FIGURE 7.**
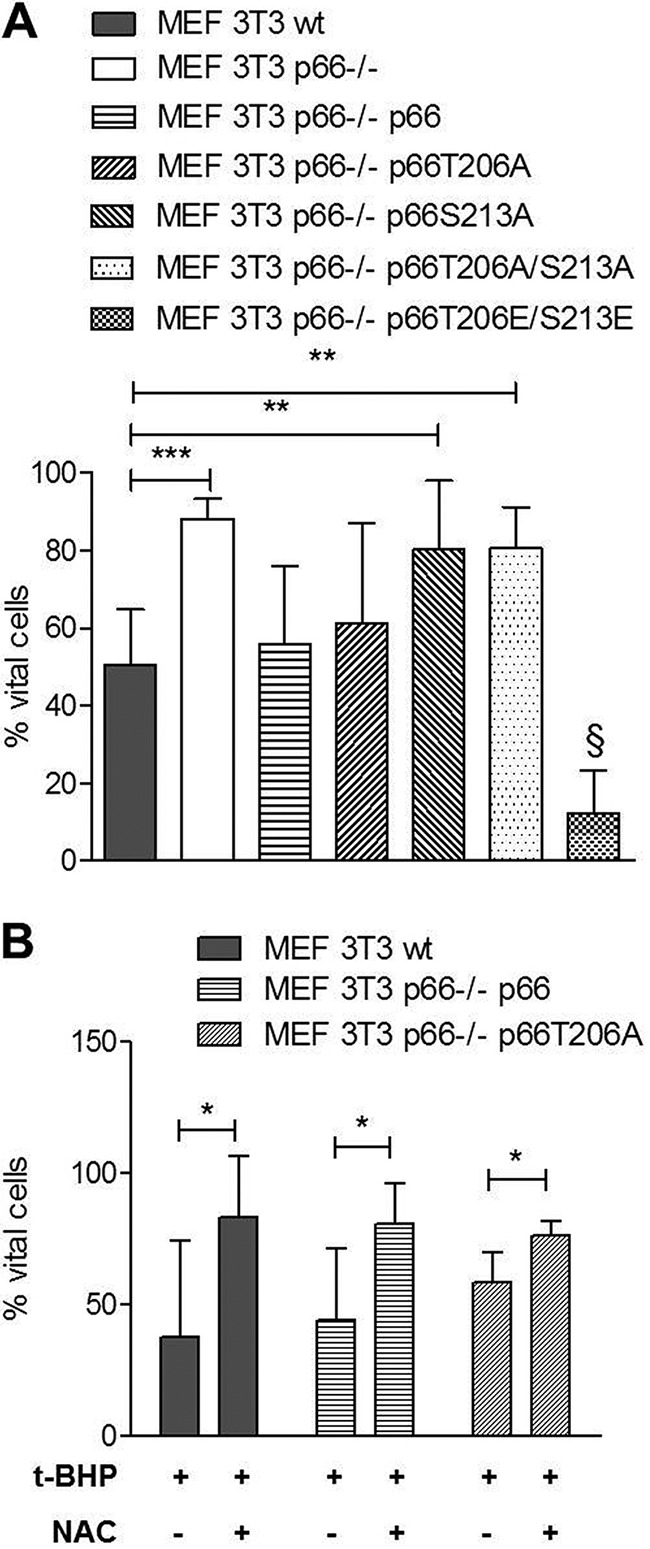
**Ser^213^ in p66 regulates ROS-induced apoptosis.** *A*, survival of MEFs expressing WT p66 or p66 mutated in Thr^206^ and/or Ser^213^ after 24-h treatment with 30 μm *t*-BHP (*n* ≥ 5). *B*, pretreatment with NAC (10 mm, 1 h) rescued MEFs expressing wild-type or Thr^206^-mutated p66 from apoptosis induced by *t*-BHP (30 μm, 24 h) (*n* ≥ 5). Statistical significances were determined using *t* test or analysis of variance (*, *p* < 0.05; **, *p* < 0.01; ***, *p* < 0.001).

Next we tested whether the ROS-deficient phenotype of cells expressing p66 mutated in Ser^213^ also resulted in increased resistance to apoptosis in response to different stress stimuli. 24-h treatment of p66^−/−^ MEFs expressing p66S213A with *t*-BHP resulted in about 80% vital cells (Annexin V- and propidium iodide-negative) similar to p66-deficient cells, in contrast to WT MEFs showing a viability of about 50% (Fig. 7*A*). We could observe the same resistance of cells expressing p66 mutated in Ser^213^ after treatment with hydrogen peroxide (H_2_O_2_), although less pronounced (data not shown). As observed for ROS production, mutation of Thr^206^ had no effect on the ability of the protein to induce cell death (Fig. 7*A*), whereas expression of p66T206E/S213E, as expected from its ROS phenotype, greatly enhanced cell death. To assure that apoptosis in response to *t*-BHP was caused by excessive ROS production, we treated cells with the anti-oxidant *N*-acetylcysteine (NAC). Pretreatment of cells with NAC rescued them from death after pro-oxidant treatment (Fig. 7*B*).

## Discussion

Excessive production or inefficient detoxification of ROS is critically involved in the initiation and progression of many pathological conditions ranging from cancer, stroke, and neurodegenerative diseases to IRI. Although the role of ROS in their development is clearly established, the use of antioxidants for the prevention of oxidative damage failed to provide significant long-term benefits (6, 46–48). Dysfunctional mitochondria cause a shortage in energy provision, enhance ROS production, and perturb mitochondrial Ca^2+^ levels. ROS damage nucleic acids, lipids, or proteins, alter the function of signaling proteins, and eventually cause cell death (49) but also lead to the activation of the inflammasome (50) or autophagy (51). p66 meets the criteria for a stress-regulated ROS-producing enzyme involved in death of cells leading to the loss or functional impairment of organs: its activation in the cytosol is triggered by cellular stress (including IR) and controlled by signaling proteins in a complex way that is not fully understood; p66 directly causes mitochondrial ROS production and cell damage; the presence of survival signals and normoxic conditions precludes p66 activation; the absence of p66 does not affect physiological ROS signaling, as evidenced by the normal development and postnatal life of p66-deficient mice (21); and a protective effect of p66 ablation was observed against IRI (27), experimental diabetic glomerulopathy (25), vascular cell apoptosis and atherogenesis induced by a high-fat diet (23), cardiomyocyte apoptosis in an experimental model of streptozotocin-induced diabetes (52), and cerebral cortex hypoxia (53). Although no inhibitors of p66 oxidoreductase activity are currently known, interfering with its activation may lead to novel therapeutic approaches to prevent or ameliorate the conditions described above.

Phosphorylation of Ser^36^ has been shown to be critical for the activation of p66 (31). However, in our experiments, we observed greatly reduced ROS production in the presence of PKC inhibitors or following PKC ablation without significant inhibition of Ser^36^ phosphorylation (Figs. 1 and 4*A*). These findings confirm the PKCβ dependence of the pro-oxidant function of p66 but strongly argue for the existence of an additional kinase responsible for Ser^36^ phosphorylation. The sequence surrounding Ser^36^ predicts phosphorylation by proline-directed kinases (54), and MAPKs have been implicated previously in the phosphorylation of Ser^36^ (33, 55, 56). Recent work by us found Ser^36^ phosphorylation by JNK1/2, which was required for ROS production and cell death induction (39). Inspection of the amino acid sequence of p66 suggested three PKC phosphorylation sites with high probability: Ser^139^, Thr^206^, and Ser^213^. All of these three sites were phosphorylated when short peptides served as substrates for recombinant PKCβ (Fig. 2*B*), whereas a peptide containing Ser^36^ only displayed background phosphorylation. Similarly, using p66 phosphorylated *in vitro* or immunoprecipitated from HEK293, we detected phosphorylation of Ser^139^ but not of Ser^36^ (Figs. 3 and 4). Phosphorylated Ser^36^ was readily detected in *in vitro* kinase assays with recombinant JNK1 (39) (Fig. 3*A*). To verify phosphorylation of Thr^206^ and/or Ser^213^ in cellular p66, a variety of MS experiments were performed, including collision-induced dissociation, higher-energy collisional dissociation, electron transfer dissociation (ETD), wide-band activation, and three-stage mass spectrometry (MS3). Moreover, immobilized metal affinity chromatography (IMAC) and TiO_2_ enrichment of phosphopeptides and digestion of proteins with two different enzymes (trypsin and lysozyme C) was carried out. In no case was phosphorylation of Thr^206^ and Ser^213^ found, which lets us speculate that the amount of p66 phosphorylated on Thr^206^ and Ser^213^ is below the detection limit. This assumption is corroborated by the demonstration that, in the *in vitro* kinase assay, we were able to detect only a small quantity of Thr(P)^206^/Ser(P)^213^ peptide (1.1%) compared with peptides phosphorylated on Ser^139^ (18%). If we predict the same ratio for our cellular assays, where, on average, we found 1.6% of the peptides to be phosphorylated on Ser^139^, detection of Thr(P)^206^/Ser(P)^213^ peptide will be impossible with the approach used currently.

Characterization of Ser^139^ confirmed its importance in the production of ROS and cell death induction following pro-oxidant treatment (Fig. 5). Although the mutation of Thr^206^ to alanine did not affect p66 function, exchange of serine in position 213 to alanine generated a mutant protein that was no longer able to respond with enhanced ROS production and cell death induction under stress (Figs. 6 and 7). However, a more complex regulation that may also involve Thr^206^ has been suggested by the demonstration that the acidic exchange of both residues rendered p66 constitutively active in terms of basal as well as inducible activity (Figs. 6 and 7). This, to our knowledge, is the first mutant of p66 displaying this property. We currently do not know which aspect in the regulation of p66 activation is affected by these mutations or whether, possibly, the intrinsic oxidoreductase activity of p66 was affected. Based on our data, we propose a model for p66 phosphorylation activation where JNK1/2 are required for the phosphorylation of Ser^36^, which may result in Pin1 binding and mitochondrial import, as reported previously for PKCβ (21). Subsequent PKCβ phosphorylation of Ser^139^, Thr^206^, and Ser^213^ is required for full activation. Which steps in the previously postulated sequence of p66 activation (57) are thereby regulated is currently unclear. The WW domain of the peptidyl-prolyl cis/trans-isomerase (PPIase) Pin1 binds phosphorylated Ser/Thr-Pro (Ser(P)/Thr-Pro) motifs, and the PPIase domain then catalyzes cis/trans-isomerization of such proline-containing peptides (58). Sequence inspection of the candidate phosphorylation sites (Fig. 2*B*) indicates that only Ser^36^ conforms to a possible Pin1 binding side. Nevertheless, we have started to address a possible role of the PKCβ phosphorylation sites identified here in Pin1 binding and mitochondrial import of p66. We can confirm the expected reduction in Pin1 binding for p66 mutated in S36A but failed to detect altered binding for the p66 Ser^139^ or p66T206A/S213A mutants, suggesting that these novel sites are not directly involved in Pin1 binding and, thus, Pin1-dependent mitochondrial translocation of p66 (data not shown). However, the whole situation may be much more complex because Pin1-mediated prolyl isomerization is a critical step in JNK activation (59). Also, evidence has been obtained that JNK1/2 bind to p66 following H_2_O_2_ treatment (39) and that JNK1/2 can translocate to the mitochondria under stress via binding to the outer mitochondrial membrane protein Sab, resulting in ROS production and cell death (60–62). It remains to be shown whether this can provide an alternative route for the mitochondrial translocation of p66.

We thus cannot exclude that these novel PKCβ phosphorylation sites are somehow involved in mitochondrial import. However, one fact that complicates the analysis of mitochondrial translocation is the observation we consistently made in various cell systems that mitochondrial p66 is present in substantial quantities already in unstimulated cells (here we also did not observe a difference in the Ala and Glu mutants studied so far) and that the mitochondrial translocation following stimulation is not immediately obvious, as *e.g.* in the case of JNK1/2 (data not shown). It may therefore very well be that it is the import of *de novo* phosphorylated protein, which may be a minor fraction, that is critical for the mitochondrial function of p66.

In summary, our experiments provide evidence for a complex mechanism regulating the activation of p66 under stress. Given the importance of p66 in the development of various disease conditions, targeting the key kinases (PKCβ and JNK1/2) involved in triggering the pro-oxidant and pro-apoptotic function of p66 may become a realistic therapeutic option.

## Experimental Procedures

### 

#### 

##### Cell Culture and Transfection

MEF 3T3 WT, p66^−/−^ (21), and HEK293 cells were cultivated in DMEM containing 10% (v/v) FCS, 200 mm l-glutamine, penicillin (100 units/ml), and streptomycin (100 μg/ml) (all from PAA Laboratories, Pasching, Austria) at 37 °C, 5% CO_2_. Stable cell lines were generated after transfection of MEF 3T3 p66^−/−^ cells with pBABEpuro p66 WT and mutant expression constructs using Lipofectamine 2000 (Invitrogen) and puromycin (Invitrogen) selection (0.4 μg/ml) for 2 weeks. HEK293 cells were transfected with 1.5 μg of pECFP-PKCα, β, or θ (laboratory of Gottfried Baier), and fluorescence was detected after 48 h by confocal microscopy as described previously (63).

For the inhibition of PKC, Gö6976 and Gö6850 were obtained from Calbiochem (Merck, Darmstadt, Germany). It has been shown previously that Gö6976 inhibits PKCα and β1 but not the Ca^2+^-independent subtypes δ, ϵ, and ζ. Gö6850 affected the activity of all these PKC isozymes with different potency (α > β1 > ϵ > δ > ζ) (64). *t*-BHP, H_2_O_2_, and TPA were obtained from Sigma-Aldrich (Vienna, Austria).

##### Site-directed Mutagenesis

Primers for mutagenesis of p66 were obtained from Eurofins Genomics (Ebersberg, Germany). Site-directed mutagenesis was performed using the QuikChange II site-directed mutagenesis kit from Stratagene (La Jolla, CA). Amino acid exchange was confirmed by DNA sequencing at the Department of Genetic Epidemiology at the Medical University Innsbruck or at Microsynth AG (Switzerland).

##### Coomassie Staining

Following electrophoresis, the gel was soaked in fixing buffer consisting of 40% (v/v) ethanol and 10% (v/v) acetic acid for 1 h. After fixation, the gel was washed for 1 h with deionized water and incubated for 2 h in Coomassie stain (Sigma) with continuous agitation. The gel was destained with deionized water.

##### MS Analyses

After the *in vitro* kinase assay, protein bands were excised from Coomassie-stained gel and digested with trypsin from porcine pancreas (Sigma-Aldrich) or Lys C (Sigma-Aldrich) as described previously (65). Tryptic digests were analyzed using an UltiMate 3000 nano-HPLC system (Thermo Scientific, Bremen, Germany) coupled to a Q Exactive Plus or Q Exactive HF mass spectrometer (Thermo Scientific) equipped with a Nanospray Flex ionization source. Settings different to Q Exactive Plus are bracketed. The peptides were separated on a homemade fused-silica microcapillary column (75 μm inner diameter × 280 μm outer diameter × 10 cm length) packed with 3-μm reverse-phase C18 material (Reprosil). Solvents for HPLC were 0.1% (v/v) formic acid (solvent A) and 0.1% (v/v) formic acid in 85% (v/v) acetonitrile (solvent B). The gradient profile was as follows: 0–2 min, 4% B; 2–55 min, 4–50% B; 55–60 min, 50–100% B, and 60–65 min, 100% B. The flow rate was 250 nl/min. The Q Exacitve Plus mass spectrometer was operating in data-dependent mode selecting the top 20 most abundant isotope patterns with a charge >1 from the survey scan with an isolation window of 1.6 *m*/*z*. Survey full-scan MS spectra were acquired from 300–1750 *m*/*z* at a resolution of 60,000 with a maximum injection time of 120 ms and automatic gain control (AGC) target 1e6. The selected isotope patterns were fragmented by higher-energy collisional dissociation with normalized collision energy of 28 at a resolution of 15,000 with a maximum injection time of 120 ms and automatic gain control target 5e5.

Data analysis was performed using Proteome Discoverer 1.4.1.14 (Thermo Scientific) with the search engine Sequest. The raw files were searched against a database (545 entries) containing the most common contaminants and p66. Precursor and fragment mass tolerance was set to 10 ppm and 0.02 Da, respectively, and up to two missed cleavages were allowed. Carbamidomethylation of cysteine, oxidation of methionine, and phosphorylation of serine, threonine, and tyrosine were set as variable modifications. The peak area of the phosphorylated and non-phosphorylated peptides was calculated by summing up the peak areas or the corresponding precursor ions.

Immunoprecipitated proteins were digested on beads with trypsin and analyzed using an UltiMate 3000 nano-HPLC system coupled via an electrospray ionization interface to an LTQ Velos mass spectrometer (Thermo Scientific). HPLC conditions were the same as with the *in vitro* kinase assay, except the particle size of the column material, which was 5-μm reverse-phase C18. The LTQ Velos mass spectrometer was operating in data-dependent mode selecting the top four most abundant isotope patterns with a charge >1 from the survey scan with an isolation window of 2.0 *m*/*z*. Survey full-scan MS spectra were acquired from 300–1800 *m*/*z* at an enhanced scan rate. Two-stage mass spectrometry (MS2) and MS3 (neutral loss from MS2) scans were fragmented by collision-induced dissociation with a normalized collision energy of 35. The settings for data analysis were the same as with the *in vitro* kinase assay, except for fragment mass tolerance, which was set to 0.8 Da. The amount of phosphorylated and non-phosphorylated peptide was calculated by the peak heights of the extracted ion chromatograms

##### ROS Measurements

Mitochondrial ROS were imaged by fluorescence microscopy after staining the cells with 100 nm MitoTracker Red CM-H_2_XROS from Molecular Probes (Invitrogen) in serum-free DMEM. For this purpose, 30,000–120,000 cells/chamber were seeded in 8-well chambered Lab-Tek coverglasses (Nalge Nunc, Rochester, NY). Staining was performed for 30 min at 37 °C. Images were taken using an Olympus IX-70 inverted microscope with an Olympus ×40 water (numerical aperture, 0.8) and an Olympus U-RFL-T mercury vapor lamp. Image acquisition was performed with a Kappa ACC1 camera and Kappa ImageBase software (Oakland, CA). For MitoTracker Red CM-H_2_XROS, a 568-nm filter was used with a light expose of 1.6 s. Gray values were measured using Scion Image software (Sigma-Aldrich, St. Louis, MO) for Windows. For every experimental condition, gray values from 100 cells were averaged. Alternatively, cellular ROS levels were determined by staining with DCF-diacetate (DCF-DA) (Sigma). Cells were loaded with 7 μm DCF-DA and incubated for 15 min at 37 °C before FACS measurement. Analysis was done using CellQuest software for FACScalibur (BD Biosciences).

##### siRNA, RNA Isolation, and Real-time PCR

ON-TARGET Plus SMARTpool mouse PRKCB1 siRNA was purchased from Dharmacon (Lafayette, CO) and transfected using Lipofectamine 2000 (Invitrogen) according to the protocol of the manufacturer. RNA isolation was performed using Qiagen RNeasy mini kit (Qiagen, Hilden, Germany), and cDNA synthesis was done using the Fermentas RevertAid first strand cDNA synthesis kit (St. Leon-Rot, Germany). PKCβ expression was determined by real-time PCR using RT^2^ real-time SYBR Green-fluorescein PCR master mix (Bio-Rad) according to the protocol provided on an iQ5 multicolor real-time PCR detection system from Bio-Rad. Using 100 nm PKCβ siRNA, on average 86% knockdown was achieved and was used for further experiments. The RT^2^ qPCR Primer Assay for Mouse PRKCB1 was ordered from Qiagen. The expression was normalized to the housekeeping gene *RSP-29*. Data analysis was performed using the 2^−ΔΔCt^ method (66). Melting curve analysis was done for quality control.

##### Immunoblotting

Protein lysates were prepared, separated, and analyzed as described previously (14). The following antibodies were used: Shc1 (610082, BD Biosciences), p66S36 (54518, Abcam, Cambridge, UK), and α-tubulin (T5168, Sigma).

##### Protein Kinase Assay

The protein kinase assay has been described previously (67). Briefly, the PKCβ-dependent phosphorylation of GST-Shc-1 peptides was measured by the incorporation of inorganic phosphate ^32^P_i_ from [γ-^32^P]ATP. The following peptides were used: Ser^36^, NH_2_-PEELPSPSASSL-COOH; Ser^139^, NH_2-_WTRHGSFVNKPT-COOH; Thr^206^, NH_2_-GAKGATRRRKPC-COOH; and Ser^213^, NH_2_-RRKPCSRPLSSI-COOH. NH_2_-QKRPSQRSKYL-COOH was used as a positive control. The level of radioactivity bound to phosphocellulose filters was counted using a Wallac MicroBeta 1450 (PerkinElmer Life Sciences). A detailed *in vitro* kinase assay protocol for phosphorylation of recombinant p66 by recombinant PKCβ has been described previously (39).

##### Immunoprecipitation

HEK293 cells were transfected with 2 μg of pBABEpuro p66 (68) using Lipofectamine (Invitrogen). 48–72 h after transfection, 300,000 or 600,000 cells were lysed in 1 ml of Nonidet P-40 lysis buffer supplemented with a protease inhibitor mixture set from Calbiochem (Merck). 40 μl of the lysate was kept as lysate control, mixed with 8 μl of 6× Laemmli buffer, and incubated for 5 min at 95 °C. Preclearing was performed with 40 μl of protein G-agarose beads from Roche, incubation for 1 h at 4 °C, and shaking at 300 rpm. Immunoprecipitation was done for 4 h with 40 μl of protein G-agarose (Roche) and 2.0 μg of Shc antibody.

##### Cell Death Assays

Apoptosis was determined using FACS after staining with Annexin V-FITC (Enzo Lifesciences, Farmingdale, NY) and propidium iodide (Carl Roth, Karlsruhe, Germany) or by counting stained (dead) and unstained (living) cells with a Neubauer counting chamber after incubation with trypan blue (Sigma-Aldrich). A detailed protocol has been described previously (39).

##### Statistics

All data are presented as mean ± S.D. Statistical analysis was done using GraphPad Prism 5 (GraphPad Software, La Jolla, CA) using *t* test or ANOVA. Significance values were designated as follows: *, *p* < 0.05; **, *p* < 0.005; ***, *p* < 0.0005.

## Author Contributions

M. H. and S. K. designed, conducted, and analyzed experiments and prepared first drafts of the manuscript and figures. M. H. and T. F. performed experiments. F. F. and G. B. conducted PKC kinase assays and provided reagents. L. K. and H. L. performed MS studies. G. B. provided expert advice on PKC signaling. J. G. and A. D. generated mutant p66 constructs and provided technical support for many procedures. M. L. and M. G. provided important tools. J. T. provided overall coordination and supervision of the study and wrote the manuscript together with S. K.

## Footnotes

3 The abbreviations used are:

IRI: ischemia/reperfusion injury
ROS: reactive oxygen species
ETC: electron transport chain
TPA: 12-*O*-tetradecanoylphorbol-13-acetate
MEF: mouse embryonic fibroblast
DCF-DA: 2′,7′-dichlorodihydroflourescence diacetate
PTB: phosphotyrosine binding
NAC: *N*-acetylcysteine
Lys C: lysozyme C
ANOVA: analysis of variance
*t*-BHP: *tert*-Butylhydroperoxid.
